# Pliocene‐Pleistocene evolution of sea surface and intermediate water temperatures from the southwest Pacific

**DOI:** 10.1002/2016PA002954

**Published:** 2016-06-30

**Authors:** Erin L. McClymont, Aurora C. Elmore, Sev Kender, Melanie J. Leng, Mervyn Greaves, Henry Elderfield

**Affiliations:** ^1^ Department of GeographyDurham UniversityDurhamUK; ^2^ Centre for Environmental Geochemistry, School of GeographyUniversity of NottinghamNottinghamUK; ^3^ British Geological SurveyNottinghamUK; ^4^ Department of Earth SciencesUniversity of CambridgeCambridgeUK; ^5^ Deceased 19 April 2016

**Keywords:** Pliocene, Pleistocene, SST, AAIW, South Pacific, DSDP

## Abstract

Over the last 5 million years, the global climate system has evolved toward a colder mean state, marked by large‐amplitude oscillations in continental ice volume. Equatorward expansion of polar waters and strengthening temperature gradients have been detected. However, the response of the mid latitudes and high latitudes of the Southern Hemisphere is not well documented, despite the potential importance for climate feedbacks including sea ice distribution and low‐high latitude heat transport. Here we reconstruct the Pliocene‐Pleistocene history of both sea surface and Antarctic Intermediate Water (AAIW) temperatures on orbital time scales from Deep Sea Drilling Project Site 593 in the Tasman Sea, southwest Pacific. We confirm overall Pliocene‐Pleistocene cooling trends in both the surface ocean and AAIW, although the patterns are complex. The Pliocene is warmer than modern, but our data suggest an equatorward displacement of the subtropical front relative to present and a poleward displacement of the subantarctic front of the Antarctic Circumpolar Current (ACC). Two main intervals of cooling, from ~3 Ma and ~1.5 Ma, are coeval with cooling and ice sheet expansion noted elsewhere and suggest that equatorward expansion of polar water masses also characterized the southwest Pacific through the Pliocene‐Pleistocene. However, the observed trends in sea surface temperature and AAIW temperature are not identical despite an underlying link to the ACC, and intervals of unusual surface ocean warmth (~2 Ma) and large‐amplitude variability in AAIW temperatures (from ~1 Ma) highlight complex interactions between equatorward displacements of fronts associated with the ACC and/or varying poleward heat transport from the subtropics.

## Key Points

- Reconstructed Tasman Sea surface and Antarctic Intermediate Water temperatures
- Long‐term cooling trends from ~3.0 to 2.6 Ma and from 1.5 Ma to present
- Complex subtropical front displacement and subantarctic cooling trends since Pliocene

## 1 Introduction

The last 5 Ma of Earth history are marked by two significant transitions that represent both a change in mean global climate state and an evolving response to external forcing by solar radiation. The onset or intensification of northern hemisphere glaciation (INHG) is usually defined at ~2.7 Ma, but occurs within a broader window of cooling and increasing continental ice [e.g., *De Schepper et al*., 2013; *Lisiecki and Raymo*, 2005; *Rohling et al*., 2014]. By ~1 Ma, further cooling and increased continental ice volume are accompanied by the emergence and then dominance of the large amplitude, asymmetric, quasi‐100 kyr glacial‐interglacial cycles (the “mid‐Pleistocene climate transition” (MPT) [*Clark et al*., 2006; *McClymont et al*., 2013; *Mudelsee and Schulz*, 1997]). Significantly, in the absence of noteworthy shifts in solar forcing driven by orbital variations, the MPT demonstrates increasing climatic sensitivity to external forcing through the Pleistocene [*Imbrie et al*., 1993; *Ravelo et al*., 2004].

Explanations for the INHG and MPT have tended to focus on the evolution of the Northern Hemisphere ice sheets [*Clark et al*., 2006; *Haug et al*., 2005], but changes to Antarctic ice sheet extent and circulation in the surrounding Southern Ocean have also been detected (reviewed by *De Schepper et al*. [2013]]. By decoupling the temperature and ice volume contributions to benthic foraminifera oxygen isotope composition (δ^18^O) in the deep northwest Pacific, *Woodard et al*. [2014] proposed that Antarctic ice volume increased from ~3.15 to 2.75 Ma, prior to INHG, and *Elderfield et al*. [2012] argued that a stepped increase in ice volume during marine isotope stages (MIS) 22–24 (~0.9 Ma) might be linked to ice sheet growth in the Ross Sea sector.

The Southern Ocean response to Pliocene‐Pleistocene climate evolution may have important impact(s) beyond the high latitudes. For example, cooling and expansion of subpolar water masses in the Subantarctic Atlantic since the Pliocene increased the meridional sea surface temperature (SST) gradients [*Martinez‐Garcia et al*., 2010] and are invoked to explain strengthened midlatitude and low‐latitude upwelling through the intensification of Hadley circulation [e.g., *Martinez‐Garcia et al*., 2010; *McClymont and Rosell‐Melé*, 2005; *Rosell‐Melé et al*., 2014]. An intensification and/or northward displacement of the Southern Hemisphere westerly wind belt since the Pliocene may also have increased deep ocean storage of CO_2_ via a strengthened biological pump [*Martinez‐Garcia et al*., 2011], and changes to the ventilation of deepwater masses in the Southern Ocean across the MPT have been linked to increased storage of CO_2_ in the abyssal and deep ocean [*Hodell and Venz‐Curtis*, 2006; *Peña and Goldstein*, 2014; *Sexton and Barker*, 2012]. Further high‐ to low‐latitude teleconnections may be provided through intermediate‐depth water masses, which form in the Southern Ocean and transport heat, salt, freshwater, and nutrients equatorward [*Lee and Poulsen*, 2008; *Loubere et al*., 2007; *Pahnke and Zahn*, 2005]. Where these intermediate waters are returned to the surface, through upwelling systems, there is the potential for water mass properties acquired in the Southern Ocean to be expressed in the tropics [*Pena et al*., 2008]. The possibility of such remote forcing complicates the interpretation of Plio‐Pleistocene cooling trends and zonal/meridional temperature gradients, since many of the continuous and orbitally resolved records of midlatitude and low‐latitude SST are from upwelling systems [*Dekens et al*., 2007; *Etourneau et al*., 2009; *Rosell‐Melé et al*., 2014]. Thus, while contraction of the subtropical gyres and expansion of subpolar waters are considered to be a key feature of Pliocene‐Pleistocene climate evolution [*Brierley and Fedorov*, 2010; *Fedorov et al*., 2015; *Martinez‐Garcia et al*., 2010], there are few data points from the Southern Hemisphere with which to test this hypothesis.

**Figure 1:**
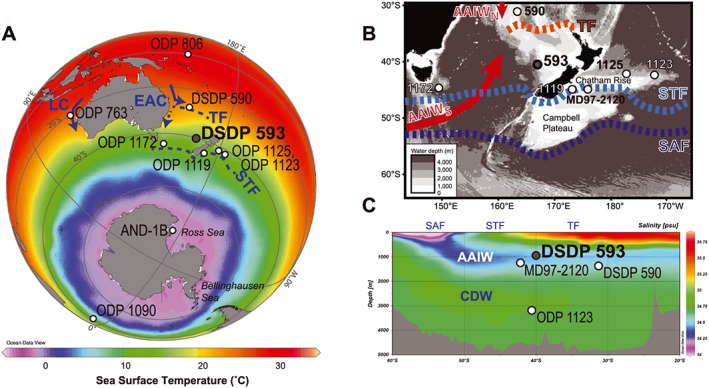
(a) Mean annual SSTs and main surface ocean circulation patterns associated with the Tasman Sea. Location of DSDP Site 593 (this study) and other sites referred to in the text are shown. TF = Tasman Front, STF = Subtropical Front, EAC = East Australian Current, LC = Leeuwin Current. (b) Tasman Sea bathymetry and major circulation patterns, adapted from *Hayward et al*. [2012]. SAF = Subantarctic Front. (c) Salinity cross section through the Tasman Sea (WOCE transect P11, longitude 155°E), indicating the low‐salinity AAIW and the position of DSDP Site 593 (this study). Data source: World Ocean Atlas 2013; Figures created using Ocean Data View [*Schlitzer*, 2002].

Here we reconstruct the Pliocene‐Pleistocene history of both surface and intermediate water properties from Deep Sea Drilling Project (DSDP) Site 593 in the Tasman Sea, southwest Pacific (Figure 1). SSTs in the Tasman Sea are sensitive to the position of the frontal systems of the Antarctic Circumpolar Current (ACC) to the south and to the extent and intensity of the subtropical gyre to the north. DSDP Site 593 has been bathed by Antarctic Intermediate Water (AAIW) through the last four glacial‐interglacial cycles [*Elmore et al*., 2015]. We present here the first continuous and orbitally resolved Pliocene‐Pleistocene reconstructions of southwest Pacific SSTs and AAIW temperatures, using the alkenone paleothermometer, U^K^_37_′ [*Müller et al*., 1998] and the Mg/Ca ratio of the benthic foraminifera *Uvigerina peregrina* [*Elderfield et al*., 2010; *Elmore et al*., 2015], respectively. We assess the hypothesized impacts of equatorward expansion of polar water masses since the Pliocene on both midlatitude SSTs and intermediate water properties and address the relative scarcity of data spanning the Plio‐Pleistocene from the Pacific sector of the Southern Ocean.

## 2 Regional Oceanography

DSDP Site 593 (40°30.47′S, 167°40.47′E, 1050 m water depth) was drilled on the Challenger Plateau of the Tasman Sea, in the southwest Pacific Ocean (Figure 1). DSDP Site 593 presently lies to the north of the Subtropical Front (STF), a complex zone delineated by large gradients in SST and salinity [*Hamilton*, 2006]. The STF separates warm, highly saline, and nutrient‐depleted subtropical surface water, sourced from the north, from cooler, lower salinity and nutrient‐rich waters sourced from subantarctic surface water and thus the Southern Ocean. To the east of DSDP Site 593, there is northward flow of subtropical surface water along the South Island of New Zealand. Modern SSTs at DSDP Site 593 range from 13.5°C (winter) to 18.5°C (summer), with an annual mean of 15°C [*Locarnini et al*., 2013]. SSTs in the Tasman Sea are considered to be more sensitive to glacial‐interglacial displacement of the STF than sites located to the east of New Zealand, where bathymetry constrains the position of both the STF and Subantarctic Front (SAF), resulting in relatively muted SST oscillations [e.g., *Carter et al*., 2004; *Hayward et al*., 2012].

DSDP Site 593 is bathed by AAIW, which is broadly characterized by low salinity (34.3–34.5 practical salinity unit (psu)), low temperatures (3.5–10°C; average density 27.1*σ*_θ_), and high dissolved oxygen (200–250 µmol kg^−1^) [*Bostock et al*., 2013; *Talley*, 1999]. Modern bottom water temperature at the site is 4–5°C, and modern salinity is ~34.5 psu. AAIW formation is complex and is closely linked to the formation of the shallower subantarctic mode waters (SAMW). AAIW formation occurs in association with the SAF, largely in the Southeast Pacific, through a range of processes including Ekman transport of Antarctic surface water (AASW), air‐sea buoyancy fluxes, and winter mixing [e.g., *Bostock et al*., 2013; *Sloyan and Rintoul*, 2001]. Intermediate‐depth circulation within the Tasman Sea includes contributions from both southern (less saline, <34.40 ± 0.0125 psu) and recirculated northern (more saline, >34.45 ± 0.0125 psu) AAIW sources, which tend to meet north of the STF [*Hamilton*, 2006]. In the modern eastern Tasman Sea, including over DSDP Site 593, a northward flow of AAIW from the Southern Ocean has been detected [*Bostock et al*., 2013; *Hamilton*, 2006].

## 3 Materials and Methods

### 3.1 DSDP Site 593: Stratigraphy and Age Model

Miocene‐Pleistocene sediments of foraminifera‐bearing nannofossil ooze extend to ~393 m depth at DSDP Site 593. Very abundant and well‐preserved benthic foraminifera are recorded [*Shipboard Scientific Party*, 1996], including the *Uvigerina* and *Planulina* species analyzed here. Sampling was guided by a low resolution but orbitally tuned stratigraphy extending back to 6.4 Ma, based on shipboard biostratigraphy and magnetostratigraphy and benthic foraminiferal δ^18^O analyses on infaunal *Uvigerina* spp. [*Head and Nelson*, 1994]. Samples were analyzed at 10–20 cm resolution in cores 593Z‐1H‐1 through 593Z‐5H‐2 (~0–36.3 m depth) and in cores 593A‐5H‐1 through 593A‐7H‐6 (36.6–64.0 m depth), to yield mean sample resolutions of ~5 kyr (0–1.5 Ma) and ~12 kyr (1.5–3.6 Ma).

A revised isotope stratigraphy (Table 1) has been generated using new analyses of benthic foraminiferal δ^18^O on the epifaunal species *Planulina wuellerstorfi* (section 3.4). The age model from 0 to 0.4 Ma has previously been published in *Elmore et al*. [2015], extended to 1.1 Ma by *Kender et al*. [2016]. The shipboard magnetostratigraphic and biostratigraphic datums [*Shipboard Scientific Party*, 1996] were re‐assigned to the GTS2012 time scale [*Gradstein et al*., 2012], although they include large depth uncertainties due to low‐resolution discrete sampling and/or difficulties identifying the presence/absence of indicator species at this site [*Shipboard Scientific Party*, 1996]. The Potaka tephra (1.0 Ma [*Shane*, 1994]) was clearly identified and centred on 21.50 m below seafloor (mbsf), and lies above a distinct benthic δ^18^O minimum, which is aligned here to MIS 31. The top of the Olduvai chron is not well represented, but the base of the Olduvai chron and the Gauss/Matuyama boundary were used to guide identification of key marine isotope stages (Table 1). It is important to note that before 1.1 Ma, glacial‐interglacial variability is detected in benthic δ^18^O but not every glacial‐interglacial cycle is clearly expressed. This poses challenges for assigning absolute isotope stages/ages to the low‐amplitude oscillations in the late Pliocene and early Pleistocene. Misalignment of isotope maxima/minima to specific glacial/interglacial stages could introduce an age uncertainty of ±40 kyr (assuming that just one obliquity‐paced cycle was missed). The age model presented here assumes that between each of the tie points outlined above, the sedimentation rate was linear. We do not seek to constrain events to the MIS scale unless they sit close to a tie‐point, and we focus instead on the longer‐term trends recorded in the data sets.

**Table 1:** Major Stratigraphic Tie Points Used in the Construction of the New Age Model for DSDP Site 593^a^

Depth (mbsf)	Age (Ma)	Tie Point	Reference
0.31	0.0159	^14^C (AMS)	*Dudley* and *Nelson* [1989]
0.81	0.088	LR04	*Elmore et al*. [2015]
1.80	0.123	LR04	*Elmore et al*. [2015]
2.31	0.138	LR04	*Elmore et al*. [2015]
3.18	0.186	LR04	*Elmore et al*. [2015]
3.86	0.237	LR04	*Elmore et al*. [2015]
4.89	0.252	LR04	*Elmore et al*. [2015]
5.28	0.295	LR04	*Elmore et al*. [2015]
5.60	0.332	LR04	*Elmore et al*. [2015]
5.80	0.341	LR04	*Elmore et al*. [2015]
7.61	0.370	LR04	*Elmore et al*. [2015]
8.07	0.421	LR04	*Kender et al*. [2016]
9.81	0.491	LR04	*Kender et al*. [2016]
10.31	0.513	LR04	*Kender et al*. [2016]
10.51	0.530	LR04	*Kender et al*. [2016]
11.01	0.584	LR04	*Kender et al*. [2016]
11.12	0.600	LR04	*Kender et al*. [2016]
12.00	0.650	LR04	*Kender et al*. [2016]
12.26	0.695	LR04	*Kender et al*. [2016]
12.81	0.706	LR04	*Kender et al*. [2016]
14.90	0.718	LR04	*Kender et al*. [2016]
15.10	0.735	LR04	*Kender et al*. [2016]
15.67	0.766	LR04	*Kender et al*. [2016]
15.88	0.790	LR04	*Kender et al*. [2016]
16.80	0.809	LR04	*Kender et al*. [2016]
17.17	0.831	LR04	*Kender et al*. [2016]
17.70	0.858	LR04	*Kender et al*. [2016]
18.10	0.874	LR04	*Kender et al*. [2016]
18.35	0.907	LR04	*Kender et al*. [2016]
18.56	0.92	LR04	*Kender et al*. [2016]
19.59	0.954	LR04	*Kender et al*. [2016]
21.20	0.987	LR04	*Kender et al*. [2016]
21.50	1.000	Potaka tephra	*Shane* [1994]
23.50	1.070	Base of Jaramillo	*Cooke et al*. [2004]
23.50	1.070	LR04 (MIS 31)	*Kender et al*. [2016]
33.33	1.778	Top of Olduvai	*Cooke et al*. [2004]
35.50	1.948	LR04 (MIS 74)	*This study*
41.90	2.438	LR04 (MIS 96)	*This study*
48.30	2.664	LR04 (MIS G2)	*This study*
56.40	3.140	LR04 (MIS KM2)	*This study*
60.50	3.295	LR04 (MIS M2)	*This study*

a Biostratigraphy and magnetostratigraphy were aligned to the GTS2012 time scale [*Gradstein et al*., 2012]. New *Planulina wuellerstorfi* δ^18^O minima and maxima [*Elmore et al*., 2015]; *Kender et al*. [2016], and this study] were visually aligned with key isotope stages in the LR04 benthic δ^18^O stack [*Lisiecki and Raymo*, 2005]. Linear sedimentation rates were assumed between all tie points.

### 3.2 Alkenone and Chlorin Analysis

Alkenones and chlorins (diagenetic products of chlorophyll [*Baker and Louda*, 1986]) were extracted from freeze‐dried and homogenized samples following the microwave‐assisted protocol of *Kornilova and Rosell‐Melé* [2003] and analyzed at Durham University. Chlorins were analyzed by UV‐vis spectrophotometry, quantified at the 410 nm and 665 nm wavelengths, and normalized for extracted sample weight [*Kornilova and Rosell‐Melé*, 2003]. The average standard deviation within samples was 0.44 units (410 nm) and 0.08 units (665 nm). Alkenones were isolated from the lipid extract using silica column chromatography, eluting with *n*‐hexane (for apolar hydrocarbons), dichloromethane (for ketones), and methanol (for polar compounds). Alkenones were quantified by Thermo Scientific Trace 1310 gas chromatograph fitted with a flame ionization detector. Separation was achieved with a fused silica column (30 m × 0.25 mm i.d.) coated with 0.25 µm of 5% phenyl methyl siloxane. The carrier gas was He. Following injection, the following oven temperature program was used: 60–200°C at 20°C/min, 200–320°C at 6°C/min, then held at 320°C for 35 min.

SSTs were calculated using the U^K^_37_′ index [*Prahl and Wakeham*, 1987] and the global mean annual SST calibration [*Müller et al*., 1998]. Alkenone concentrations were calculated with reference to the relative response of an internal standard (2‐nonadecanone, Sigma‐Aldrich) and the extracted dry weight of sediment. We were unable to correct the alkenone and chlorin concentrations to mass accumulation rates, due to the very low resolution shipboard porosity and wet density measurements [*Shipboard Scientific Party*, 1996]. However, no changes in sedimentation rates were associated with shifts in alkenone or chlorin concentrations, so we interpret the data here as indicative of organic matter flux to the seafloor at the site.

### 3.3 Benthic Foraminiferal Mg/Ca Analysis

The detailed methods applied here have been published previously [*Elmore et al*., 2015]. Briefly, approximately 10 individuals of visually well‐preserved *Uvigerina peregrina* were picked from the >250 µm fraction for elemental analysis and prepared following the sequential rinsing and oxidative cleaning protocol of *Barker et al*. [2003]. Mg/Ca ratios were measured by inductively coupled plasma optical emission spectrometry (Varian, Vista) at the Godwin Laboratory for Palaeoclimate Research at Cambridge University. Instrumental precision was ±0.51%, calculated by replicate analyses of a standard solution with Mg/Ca of 1.3 mmol/mol. Interlaboratory studies have established the accuracy of Mg/Ca determinations [*Greaves et al*., 2008; *Rosenthal et al*., 2004], confirmed here by replicate analysis of an interlaboratory comparison standard JCt‐1 (mean Mg/Ca 1.265 ± 0.011 mmol/mol), consistent with the reported mean Mg/Ca of 1.289 ± 0.045 mmol/mol [*Hathorne et al*., 2013]. Fe/Ca and Mn/Ca were measured simultaneously and record values of less than 0.06 mmol/mol and 0.07 mmol/mol, respectively, for all analyses from DSDP Site 593, indicating insignificant contamination by clay minerals or diagenetic coatings [*Barker et al*., 2003].

Foraminifera Mg/Ca ratios (Mg/Ca_test_) are a function of both temperature and the Mg/Ca ratio of seawater (Mg/Ca_sw_), and the relationship between Mg/Ca_test_ and Mg/Ca_sw_ is both nonlinear and shows variability between species (see discussion by *Evans and Müller* [2012]). Given the residence times of Mg (~14 Ma) and Ca (~1 Ma), the impact of changing Mg/Ca_sw_ on ocean temperature reconstructions is most important for pre‐Pleistocene sequences [*Evans and Müller*, 2012; *Medina‐Elizalde et al*., 2008]. During the Pleistocene, intermediate water temperature (IWT) can be calculated using the *U*. *peregrina* calibration of *Elderfield et al*. [2010]:

(1) $$\mathrm{Mg}/{Ca}_{\text{test}}=\;1.0\;+\;0.1\;\times \;IWT$$

Recent studies have indicated that Pliocene Mg/Ca_sw_ was lower than modern, and thus, a correction should be applied to Mg/Ca‐temperature time series [*Medina‐Elizalde et al*., 2008; *O*'*Brien et al*., 2014]. Applying such a correction is not straightforward, however, since a temporally well‐resolved and coherent picture of Mg/Ca_sw_ in the Pliocene is not yet available, but rather a range of values have been proposed [*Fantle and DePaolo*, 2006; *O'Brien et al*., 2014]. A minimal Pliocene correction (<1°C) has also been advocated, based on considerations of warm pool properties and comparison of single‐site, multiproxy SST reconstructions [*Fedorov et al*., 2015]. To test the impact of evolving Mg/Ca_sw_ on our estimates of IWT, we follow the approach of *Evans and Müller* [2012] and *Woodard et al*. [2014] to modify equation (1):

(2) $$\mathrm{Mg}/{Ca}_{\text{test}}=\;\left(1.0\;+\;0.1\;\times \;IWT\right)\;\times \;\left[{\left(\mathrm{Mg}/{{Ca}_{sw}}^{t=t}\right)}^{H}/{\left(\mathrm{Mg}/{{Ca}_{sw}}^{t=0}\right)}^{H}\right]$$

where *t* = 0 is modern, *t* = *t* is the given sample age, and *H* is the species‐specific power component of the relationship between Mg/Ca_test_ and Mg/Ca_sw_. In the absence of a *U*. *peregrina* value for *H*, we adopt the approach of *Woodard et al*. [2014] and use a conservative estimate of 0.41 [*Delaney et al*., 1985]. We apply a suite of measured, modeled, and back‐calculated (outlined in *O'Brien et al*. [2014]) estimates of Mg/Ca_sw_ to generate a range of possible corrections. As discussed below, these Mg/Ca_sw_ corrections raise IWTs during the Pliocene by up to 5°C, although the overall trends and timings of events are independent of the correction applied.

### 3.4 Foraminiferal Stable Isotopes

Previous studies at DSDP Site 593 had analyzed the oxygen and carbon isotope composition of both planktonic (*Globigerina bulloides*) and infaunal benthic (*Uvigerina* spp.) foraminifera extending to the Miocene [*Cooke*, 2003; *Head and Nelson*, 1994]. Here we present new δ^18^O and δ^13^C analyses of the epibenthic foraminifera *P*. *wuellerstorfi* to 64 mbsf, since this species precipitates calcite in isotopic equilibrium with ambient seawater, whereas isotopic fractionation during calcite precipitation in *U. peregrina* may be affected by other factors including pore water pH and organic carbon flux to sediments [*Elmore et al.*, 2015; *Marchitto et al*., 2014; *Zahn et al*., 1986].

Approximately four individuals of *P. wuellerstorfi* were picked from the >250 µm fraction. Samples spanning 0–1.5 Ma were analyzed using an IsoPrime dual inlet mass spectrometer plus Multiprep device at the Natural Environment Research Council Stable Isotope Facility (BGS); samples spanning 1.5–3.5 Ma were analyzed at the Godwin Laboratory for Palaeoclimate Research at Cambridge University. Stable isotopic compositions are reported using standard delta notation, δ^13^C and δ^18^O, representing the deviation in ^13^C/^12^C and ^18^O/^16^O from the Vienna Peedee belemnite standard and are reported in units of per mil (‰). Analytical reproducibility of the in‐house calcite standards was less than ±0.1‰ for both δ^13^C and δ^18^O at both laboratories.

## 4 Results

### 4.1 Alkenone SSTs and Concentrations

Overall, the alkenone concentrations at DSDP Site 593 were low (<0.4 µg g^−1^), despite the dominance of nannofossils in the core lithology [*Shipboard Scientific Party*, 1996]. In 137 samples alkenones were not detected and/or their concentrations were too low to quantify the U^K^_37_′ index with confidence. Although previous work in the midlatitude to high latitude of the Southern Hemisphere has detected the subpolar water mass indicator (the C_37:4_ alkenone) during late Pleistocene glacial stages [e.g., *Ho et al*., 2012; *Martinez‐Garcia et al*., 2010], this alkenone was rarely detected at DSDP Site 593, consistent with the relative warmth of the SSTs throughout (generally >8°C).

**Figure 2:**
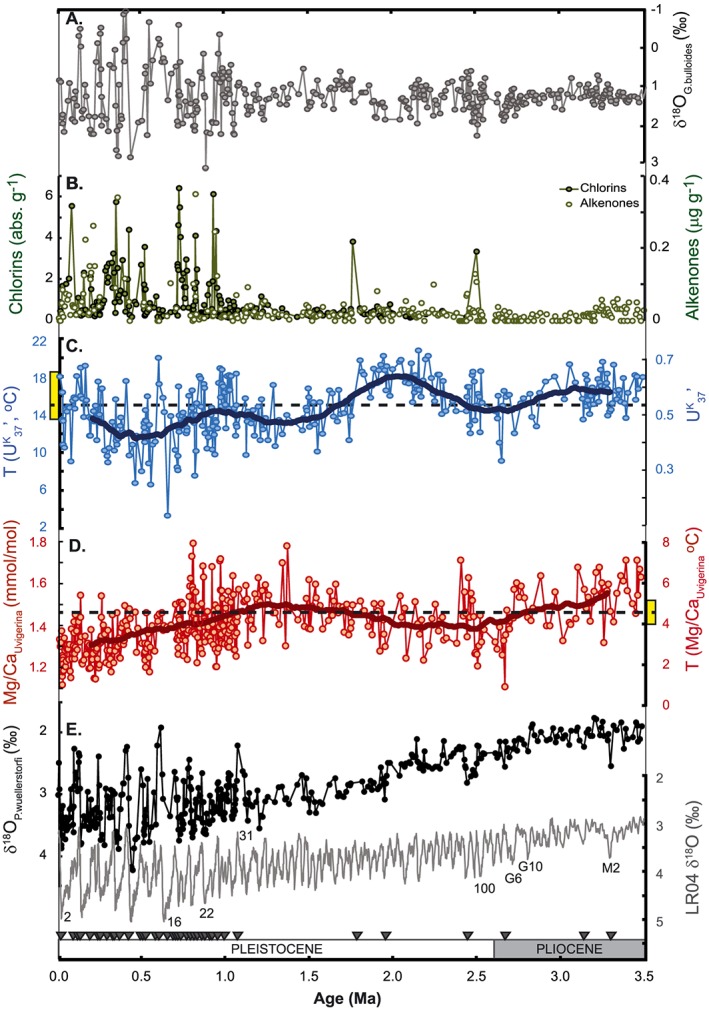
Pliocene‐Pleistocene data from DSDP Site 593. (a) δ^18^O in *G*. *bulloides*, from *Cooke* [2003]; (b) alkenone and chlorin concentrations (this study); and (c) alkenone unsaturation index (U^K^_37_′) and calculated SSTs (this study, blue dots), with 400 kyr running mean (thick blue line). The modern annual mean SST is delineated by the horizontal dashed line, and the modern annual range by the yellow box on the temperature *y* axis. (d) Mg/Ca_*U*.*peregrina*_ ratios and reconstructed intermediate water temperatures, uncorrected for Mg/Ca_sw_ evolution (this study, red dots), with 400 kyr running mean (thick red line). The modern mean and range for the Tasman Sea are shown as in Figure 2c. (e) δ^18^O_*P*.*wuellerstorfi*_ (this study, black) and the benthic foraminiferal δ^18^O stack of *Lisiecki and Raymo* [2005; grey]. Age model tie points (Table 1) are indicated by triangles, and key MIS are labeled.

A large range in U^K^_37_′‐SSTs is recorded at DSDP Site 593 over the Pliocene and Pleistocene (3.3–20.7°C; Figure 2). During the late Pliocene and early Pleistocene, glacial‐interglacial variability of 4–6°C is recorded, with minima close to modern winter (13.5°C) and maxima exceeding modern summer (18.5°C). A long‐term cooling trend from 3.1 Ma (6°C Myr^−1^) culminates in culminates in a pronounced cooling event at 2.65 Ma, which reduces SSTs to values subsequently only recorded during the late Pleistocene glacial stages (~11°C). After 2.65 Ma SSTs warm (6.2°C Myr^−1^) toward an interval of sustained high mean SSTs (18°C) between 2.3 and 1.8 Ma, with SSTs persistently exceeding both the modern annual average and late Pliocene values. From 1.8 Ma there is a second cooling trend (7.5°C Myr^−1^) until ~1.3 Ma, and a final cooling trend occurs from 0.9 to 0.6 Ma (7.5°C Myr^−1^). From 1.1 Ma the amplitude of the glacial‐interglacial oscillations in SST increases to 8–12°C, with interglacial maxima (17–20°C) comparable to, or exceeding modern summer values, and glacial minima (3–12°C) lying below those of modern winter (Figure 2).

Alkenone concentrations at DSDP Site 593 fluctuate on orbital timescales across a range from 0 to 0.35 µg g^−1^ (Figure 2). Between 3.0 and 2.5 Ma alkenone concentrations are particularly low (<0.025 µg g^−1^), but increased variability is recorded after 2.5 Ma (0–0.013 µg g^−1^) and after 1.0 Ma (0–0.35 µg g^−1^). The chlorin data set does not extend to the Pliocene, but where the chlorin and alkenone data sets overlap (1.5–0 Ma), a similar overall pattern is expressed, with increased variability after 1.0 Ma (Figure 2), and an overall increase in organic matter flux from the Pliocene to present.

### 4.2 Mg/Ca Intermediate Water Temperatures (IWT)

The Mg/Ca*_U.peregrina_* ratios at DSDP Site 593 range from 1.01 to 1.8 mmol mol^−1^, equivalent to Mg/Ca_sw_‐uncorrected IWTs of 0.97 to 7.9°C (Figure 2). In general, glacial‐interglacial temperature fluctuations of 3–4°C amplitude are recorded. The reduced amplitude variability between 1.5 and 2.5 Ma may reflect the lower temporal resolution of the record as a result of very low concentrations of *Uvigerina*. IWT from uncorrected Mg/Ca*_U.peregrina_* shows subtle long‐term trends: gradual cooling from a Pliocene average of ~5.2°C begins ~3.1 Ma, a relatively abrupt and pronounced cooling develops from 2.7 Ma (to 0.9°C), and a small (~1°C) warming occurs from 2.0 to 1.3 Ma. After 1.3 Ma, there is an increase in interglacial maxima and a progressive decline in glacial maxima [*Kender et al*., 2016], superimposed upon a monotonic cooling of ~2°C toward the present day. From 0.8 Ma, interglacial maxima cool to align with modern AAIW temperatures of ~4°C [*Elmore et al*., 2015], reducing the orbital‐scale variability to c.4°C.

The incorporation of a number of trace elements into benthic foraminifera calcite can be influenced by carbonate ion saturation (Δ[CO_3_^2−^]). This has enabled reconstructions of past Δ[CO_3_^2−^] using both B/Ca and Mg/Ca ratios in *P. wuellerstorfi* [e.g., *Rae et al*., 2011; *Elmore et al*., 2015; *Kender et al*., 2016]. We do not find any relationship between the ratios of Mg/Ca*_U.peregrina_*, Mg/Ca*_P.wuellerstorfi_*, nor B/Ca*_P.wuellerstorfi_* at DSDP Site 593 over the last 1.1 Ma [*Elmore et al*., 2015; *Kender et al*., 2016], confirming previous work which has shown a minimal impact of Δ[CO_3_^2−^] on *U. peregrina* Mg/Ca ratios, and a stronger relationship to bottom water temperatures [e.g., *Elderfield et al*., 2010].

**Figure 3:**
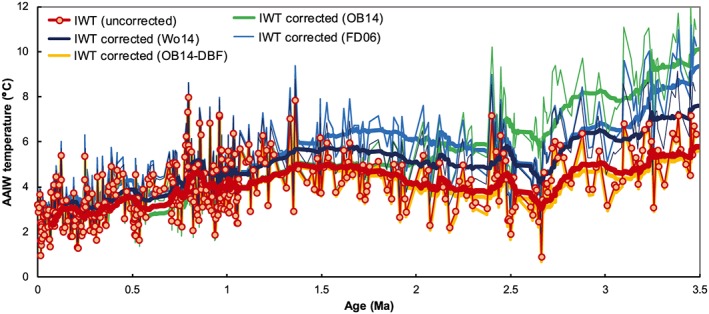
Comparison of the impact of Pliocene‐Pleistocene seawater Mg/Ca (Mg/Ca_sw_) corrections on reconstructed Antarctic Intermediate Water (AAIW) temperatures at DSDP Site 593. Uncorrected Mg/Ca applies only the Mg/Ca _*U*.*peregrina*_ temperature calibration of *Elderfield et al*. [2010]. OB14‐DBF applies the third‐order polynomial fit of *O'Brien et al*. [2014]; Wo14 applies a linear fit from a conservative estimate of Pliocene Mg/Ca_sw_ by *Woodard et al*. [2014]; OB14 applies a back‐calculated Mg/Ca_sw_ based on multiproxy SST estimates [*O'Brien et al*., 2014]; FD06 applies a modeled Mg/Ca_sw_ evolution, which allows for variable weathering fluxes to the ocean [*Fantle and DePaolo*, 2006; *Medina‐Elizalde et al*., 2008]. Original data (thin lines) and 25 point running means (thick lines) are shown for all time series.

The absolute values of Pliocene IWT (and thus the magnitude of the Pliocene‐Pleistocene cooling trend) are impacted by Mg/Ca_sw_ corrections, which elevate mean Pliocene IWTs from being comparable to modern (within 1°C) to between 2 and 5°C higher (Figure 3). There remains debate and uncertainty about the magnitude and timing of Mg/Ca_sw_ corrections, and how they should be applied to the benthic foraminifera temperature calibration [*Dekens et al*., 2008; *Medina‐Elizalde et al*., 2008; *Woodard et al*., 2014]. *Woodard et al*. [2014] showed that Mg/Ca_sw_ corrections at deepwater sites Ocean Drilling Program (ODP) 1208 and 607 gave unrealistic Pliocene temperatures in the water mass source regions. The data from DSDP Site 593 do not provide similar constraints on the feasibility of the different Mg/Ca_sw_ corrections. Late Pliocene interglacial maxima in uncorrected IWT (6–7°C) fall within the range simulated for intermediate‐depth waters for the southwest Pacific between 3.1 and 3.3 Ma (broadly 500–1200m, 4–8°C) [*Dowsett et al*., 2009], whereas the corrected values exceed the modeled range. However, the full range of Mg/Ca_sw_‐corrected IWT all remain below the SSTs recorded in the likely source region of AAIW, the subantarctic ACC (ODP 1090, 10–19°C) [*Martinez‐Garcia et al*., 2010]. As Mg/Ca_sw_ evolves toward the modern value, the offsets between uncorrected and corrected data decrease to less than 1°C by 1 Ma, making the correction smaller than analytical uncertainty, and thus unnecessary for the middle and late Pleistocene.

### 4.3 Foraminiferal Stable Isotopes

The planktonic δ^18^O record from DSDP Site 593 was previously reported [*Cooke*, 2003; *Head and Nelson*, 1994]. Overall, the δ^18^O*_G.bulloides_* data oscillate around a stable Pliocene‐Pleistocene mean of approximately +1.0‰. A large increase in orbital‐scale variability toward the present day occurs at 1.1 Ma, from < +1.27‰ to > +2.5‰ (Figure 2). Accounting for the Pliocene‐Pleistocene trends in SST at DSDP Site 593 and the overall increase in continental ice volume over the same time window [*Rohling et al*., 2014], these trends indicate an overall reduction in sea surface salinity at DSDP Site 593 since the Pliocene.

**Figure 4:**
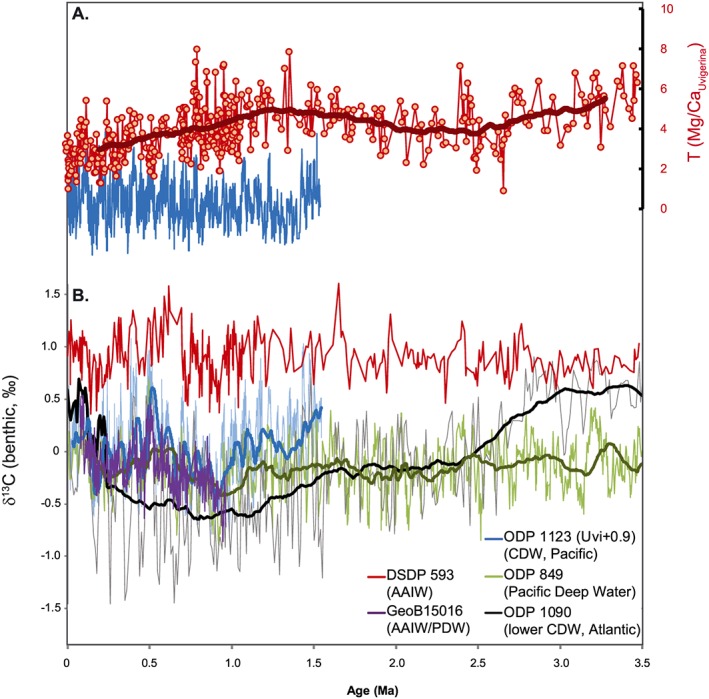
Comparison of benthic foraminifera temperature and δ^13^C data from DSDP Site 593 with published data sets. (a) DSDP Site 593 Mg/Ca_*U*. *peregrina*_ (this study), uncorrected for Mg/Ca_sw_ evolution, compared to Mg/Ca_*U*.*peregrina*_ from ODP Site 1123 [*Elderfield et al*., 2012]. (b) Benthic foraminiferal δ^13^C from DSDP Site 593 (this study). GeoB15016 [*Martínez‐Méndez et al*., 2013] is bathed by AAIW during glacial maxima; ODP Sites 1123 [*Elderfield et al*., 2012] and Site 849 [*Mix et al*., 1995] are bathed by Pacific Deep Water. ODP Site 1090 is bathed by lower CDW within the Atlantic basin [*Hodell and Venz‐Curtis*, 2006]. Smoothing at ODP Sites 849 and 1090 by *Hodell and Venz‐Curtis* [2006]. For site details see Table 2.

Benthic foraminiferal δ^18^O_*P*.*wuellerstorfi*_ from DSDP Site 593 increases from the Pliocene to present (Figure 2), consistent with global trends of cooling and increasing continental ice volume [*Lisiecki and Raymo*, 2005; *Rohling et al*., 2014]. Between 2.5 and 2.4 Ma there is a sustained but temporary increase in δ^18^O*_P.wuellerstorfi_*, and from 1.0 Ma an increase in variability is observed. Long‐term trends are less clearly defined in benthic foraminiferal δ^13^C*_P.wuellerstorfi_*, which oscillates around average values of +0.8 to +0.9‰ (Figure 4). Before 3 Ma, the amplitude of δ^13^C*_P. wuellerstorfi_* variations is relatively muted (<0.4‰); after 3 Ma, oscillations with an amplitude >0.45‰ are recorded.

## 5 Discussion

### 5.1 Pliocene‐Pleistocene Climate Evolution in the Eastern Tasman Sea

#### 5.1.1 Surface Ocean Circulation

Remarkably different signatures of Pliocene‐Pleistocene temperature evolution are recorded between the U^K^_37_′ and Mg/Ca*_U.peregrina_* data from DSDP Site 593, despite the hypothesis that both relate to high‐latitude climate changes via connections to the ACC. Both data sets show elements of the typical trend of combined overall cooling and increasing orbital‐scale variability toward the present day [*Fedorov et al*., 2015, 2013; *McClymont et al*., 2013], but SSTs are warmest in the early Pleistocene and IWTs show reduced variability in the late Pleistocene (Figure 2).

**Figure 5:**
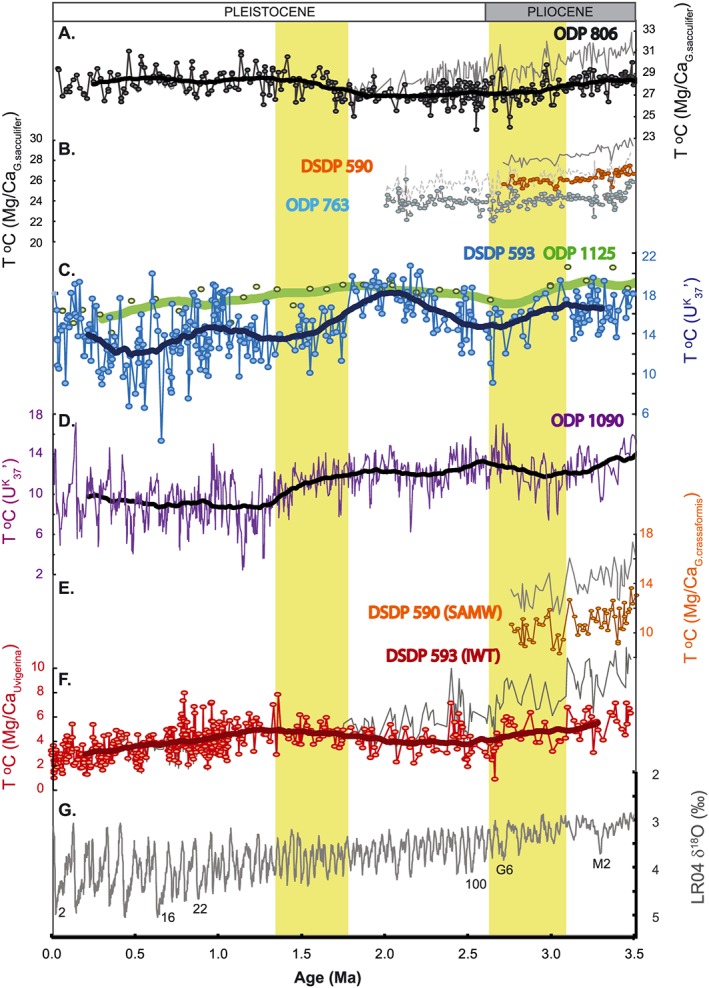
Comparison of DSDP Site 593 SSTs and IWTs (this study) to Pliocene‐Pleistocene temperature records from the Western Pacific Ocean, South‐eastern Indian Ocean, and Subantarctic Atlantic Ocean. Site locations are shown on Figure 1. (a) West Pacific Warm Pool SSTs (ODP Site 806) [*Wara et al*., 2005]; (b) Leeuwin Current region SSTs (ODP Site 763A) and northern Tasman Sea SSTs (ODP Site 590B) [*Karas et al*., 2011a]; (c) SSTs from two sites presently situated north of the STF, in the Tasman Sea (DSDP site 593, this study) and on the Chatham Rise (ODP Site 1125) [*Fedorov et al*., 2015]; (d) Subantarctic Atlantic SSTs (ODP Site 1090, between the STF and the SAF) [*Martinez‐Garcia et al*., 2010]; (e) SAMW temperatures from DSDP Site 590A [*Karas et al*., 2011b]; and (f) AAIW temperatures from DSDP site 593 (this study). (g) The global benthic foraminiferal δ^18^O stack is shown for reference [*Lisiecki and Raymo*, 2005]. For those records generated using foraminifera Mg/Ca, the uncorrected (coloured lines, symbols) are presented alongside the results of the largest seawater correction, from OB14 (Figure 3; thin grey lines for each site). All sites have benthic foraminiferal δ^18^O stratigraphies, except ODP Site 1125, which is based on a low‐resolution biostratigraphic age model [*Fedorov et al*., 2015]. All SST time series are shown to the same vertical scale. The 400 kyr running means are shown for all sites which span the Pliocene and Pleistocene (thick lines).

**Figure 6:**
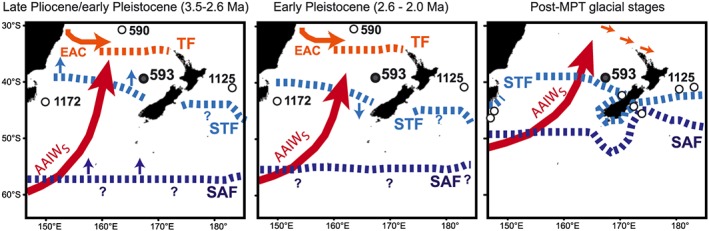
Schematic of potential changes to surface and intermediate water circulation in the southwest Pacific since the late Pliocene. Sites which inform the conceptual framework for each time interval are shown. (a) Late Pliocene, with amplified EAC and poleward displacement of the Tasman Front (from DSDP Site 590B) and equatorward displacement of the STF (from DSDP Site 593 and ODP Site 1172) relative to modern, whereas warmer IWTs than modern and reduced sea ice extent [*Barron*, 1996a, 1996b] suggest an overall poleward displacement of the SAF. Cooling in SSTs and IWT at DSDP Site 593 from ~3 Ma suggests ongoing subantarctic cooling and/or equatorward migration of the STF and SAF (blue arrows). (b) Early Pleistocene, with the Tasman Front still displaced poleward and a strong EAC (DSDP Site 590B). Cooling in SSTs and IWTs at DSDP Site 593 indicates poleward migration of the STF and SAF, but the STF remains north of ODP Site 1172. (c) Late Pleistocene glacial stages, which are marked by large equatorward displacements of the STF and SAF, as well as increased bathymetric control over front positions to the east of New Zealand (constrained by multiple sites in *Hayward et al*. [2012] and *Sikes et al*. [2009], site numbers not shown here). The TF also migrated northward but some influence of subtropical water to the northern Tasman Sea is hypothesized (orange arrows [*Hayward et al*., 2012]). For the modern positions of the fronts please refer to Figure 1.

During the Pliocene and Pleistocene, both the orbital‐scale oscillations and longer‐term trends in SSTs at DSDP Site 593 are interpreted as evidence for varying influences of subtropical (warm) and subantarctic (cold) waters in the southern Tasman Sea. Before 2.7 Ma, the warmer‐than‐present SSTs and overall low alkenone concentrations suggest that the STF lay to the south of DSDP Site 593. These conditions are coeval with high abundances of nannofossil species characteristic of modern surface waters to the south of the STF (e.g., *Coccolithus pelagicus* and *Calcidiscus leptoporus*) being recorded at ODP Site 1172 in the southwest Tasman Sea (44°57′S; Figures 1 and 6) [*Ballegeer et al*., 2012]. Taken together, these results suggest that the late Pliocene STF was positioned between DSDP Site 593 and ODP 1172 (between 40 and 44°S), representing a relatively minor but equatorward displacement compared to modern (a maximum of 4° latitude). The 400 kyr running mean in DSDP Site 593 SSTs are ~2°C lower than an alkenone SST record from ODP Site 1125 (Figures 5 and 6) [*Fedorov et al*., 2015]. ODP Site 1125 is located to the east of New Zealand but in an equivalent modern position, north of the STF and influenced by warm surface waters from the northern Tasman Sea (Figure 1). The SST offset may in part reflect the low‐resolution (~100 kyr) sampling at ODP Site 1125, since there is some overlap with DSDP Site 593 maxima in the original data (Figure 5), or it could indicate that DSDP Site 593 was closer to the STF than ODP Site 1125 in the Pliocene (Table 2).

**Table 2:** Core Sites Discussed in the Main Text and Shown on Figure 1

Site	Lat/Long	Water Depth (m)	Reference
593	40°30′S, 167°40′E	1068	This study
590B	31°10′S, 163°22′E	1308	*Karas et al*. [2011a]
MD97‐2120	45°32′S, 174°56′E	1210	*Pahnke and Zahn* [2005]
1172	44°57′S, 149°55′E	2620	*Ballegeer et al*. [2012]
1119	44°45′S, 172°24′E	395	*Carter et al*. [2004]
1123	41°47′S, 171°30′W	3290	*Elderfield et al*. [2012], *Elmore et al*. [2015]
763A	20°35′S, 112°13′E	1367	*Karas et al*. [2011b]
806	0°19′N, 159°22′E	2532	*Wara et al*. [2005]
849	0°11′N, 110°31′W	3850	*Mix et al*. [1995], *Hodell and Venz‐Curtis* [2006]
1090	42°55′S, 8°54′E	3702	*Hodell and Venz‐Curtis* [2006], *Martinez‐Garcia et al*. [2010]
AND‐1B	77°53′S, 167°05′E	936	*McKay et al*. [2012]

The long‐term surface cooling and increased export productivity (from alkenone concentrations) at DSDP Site 593 since the Pliocene is consistent with an increasing influence of subantarctic waters and/or reduced influence of tropical waters to the southern Tasman Sea, although there is significant complexity and variability within this trend. From 3.1 Ma, synchronous surface cooling at DSDP Site 593 and ODP Site 1125 (Figure 5) occurs with increased STF nannofossil indicators at ODP Site 1172 [*Ballegeer et al*., 2012], suggesting that the STF migrated northward. This occurs when the continued restriction of the Indonesian throughflow from 3.3 Ma [*Karas et al.*, 2011b] would be expected to strengthen the EAC and thus poleward heat transport to the Tasman Sea [*Lee et al*., 2002]. At DSDP Site 590B, planktonic foraminifera Mg/Ca confirm relatively warm SSTs and a reduced temperature gradient to the West Pacific Warm Pool developing from 3.5 Ma (Figure 5b), interpreted to reflect a strong EAC influence to the northern Tasman Sea as the Indonesian gateway becomes increasingly restricted [*Karas et al*., 2011a]. Thus, the SST cooling at DSDP site 593 from 3.1 Ma is unlikely to reflect changes in the EAC, supporting our interpretation of the surface cooling as being related to the position of the STF.

The subsequent warming, from 2.65 Ma toward the early Pleistocene SST maxima at 2 Ma, would therefore reflect a southward displacement of the STF and increased subtropical surface waters to the southern Tasman Sea. ODP Site 1125 also records the early Pleistocene warming, and the cooling trend after 1.8 Ma, but the amplitude of the signal is muted compared to DSDP Site 593 (Figure 5). This might in part reflect sampling resolution, or the bathymetric control of the migration of the STF (and SAF) by the Chatham Rise and Campbell Plateau [*Hayward et al*., 2012]. Thus, as observed during late Pleistocene glacial‐interglacial cycles [*Hayward et al*., 2012], SSTs in the Tasman Sea become more sensitive to STF migration than sites to the east; Figure 5 suggests that this situation developed at least from the early Pleistocene.

After 1.0 Ma, large‐amplitude glacial‐interglacial SST variations develop. SST minima are broadly associated with alkenone and chlorin concentration maxima, consistent with previous suggestions of an increased influence of subantarctic waters and equatorward displacements of the STF in the Tasman Sea during glacial stages [*Hayward et al*., 2012; *Kender et al*., 2016; *Nürnberg and Groeneveld*, 2006]. Although the U^K^_37_′ index is calibrated to mean annual SST [*Müller et al*., 1998], seasonality in coccolithophore production has been considered as a potential influence over reconstructed absolute SSTs, especially where multiproxy analyses have been performed [*Sikes et al*., 2009]. In an assessment of globally distributed sediment traps, *Rosell‐Melé and Prahl* [2013] noted that despite highly variable seasonal patterns of alkenone flux, the sedimentary alkenone signal still closely resembled the mean annual SST calibration. However, in two sites in the southwest Pacific close to the STF, a cold bias in the sediment trap alkenone SST was determined. The authors did not link this bias specifically to seasonality, since the season of maximum production was different between sites, but instead considered that the proximity to the hydrographic fronts may play a role, albeit unexplained at present [*Rosell‐Melé and Prahl*, 2013]. If proximity to the STF does lead to a cold bias in alkenone SSTs at DSDP site 593, then the glacial‐stage cooling of the late Pleistocene may have been amplified by the northward migration of the STF. However, this interpretation contrasts with multiproxy analyses of sites lying close to the STF across the last glacial cycle, where alkenone SSTs were warmer than planktonic foraminifera assemblages and linked to summer alkenone production [*Sikes et al*., 2009]. Furthermore, our reconstructed glacial‐interglacial cycles in SST are comparable in amplitude (8–12°C) to late Pleistocene 100 kyr cycles recorded in several Tasman Sea sites using a variety of proxies [e.g., *Hayward et al*., 2012; *Nürnberg and Groeneveld*, 2006; *Nürnberg et al*., 2004; *Pelejero et al*., 2006]. The absolute SSTs at DSDP Site 593 since 1 Ma are also comparable to those recorded in sites which presently sit north of the STF [*Hayward et al*., 2012] and warmer than those situated close to or to the south of the modern STF [*Hayward et al*., 2012; *Pahnke et al*., 2003]. The data from DSDP site 593 are thus consistent with the regional‐scale evidence for equatorward displacements of the STF during glacial stages, which became particularly pronounced from 1 Ma.

#### 5.1.2 Intermediate Water Circulation

Our benthic foraminifera data indicate long‐term and glacial‐interglacial variations in intermediate‐depth ocean temperatures through the Pliocene and Pleistocene. Several mechanisms could account for these patterns at our site: shifting water mass boundaries, a change in the relative contribution of different sources of intermediate waters, or changes to conditions in the region of intermediate water mass formation.

Although large changes in intermediate‐depth water temperatures could be driven by displacement of water mass boundaries, we do not think that this accounts for the trends observed here. δ^13^C_*P*.*wuellerstorfi*_ oscillates between +0.5 and +1.5‰ throughout, without long‐term trends that might reflect a change in water mass source (Figure 4). We recognize that δ^13^C_*P*.*wuellerstorfi*_ can also reflect changes in organic matter flux to the seafloor [*Mackensen et al*., 1993], which can limit its strength as a water mass proxy, although there is no clear response in δ^13^C_*P*.*wuellerstorfi*_ to the increased export productivity indicated by the chlorin and alkenone accumulation rates after 1.1 Ma (Figures 2 and 4). No associated increase in mean or interglacial δ^13^C*_P.wuellerstorfi_* is observed, which might link an increase in SAMW depth to warm IWTs [e.g., *Lynch‐Stieglitz et al*., 1994], although the processes of SAMW and AAIW formation (and their properties) are closely linked [*Hartin et al*., 2011; *Sloyan and Rintoul*, 2001]. The lower boundary of AAIW, with upper CDW, has shoaled in the Tasman Sea and at Chatham Rise during late Pleistocene glacial stages [*Elmore et al*., 2015; *Pahnke and Zahn*, 2005; *Ronge et al*., 2015]. However, we have shown previously that Mg/Ca*_U.peregrina_* and δ^13^C*_P.wuellerstorfi_* at DSDP Site 593 remained offset from upper CDW throughout the last four glacial cycles, confirming that AAIW continued to bathe the site [*Elmore et al*., 2015]. The offset between DSDP Site 593 and lower CDW is maintained in both Mg/Ca*_U.peregrina_* and δ^13^C*_P.wuellerstorfi_* over the last 1.5 Myr (ODP Site 1123) [*Elderfield et al.,* 2012] and into the Pliocene (ODP Site 849; Figure 4) [*Mix et al.*, 1995].

AAIW properties in the modern Tasman Sea reflect variable contributions of the northern and southern sourced AAIW (AAIW_N_ and AAIW_S_; Figure 1b)[*Bostock et al*., 2004]. At present, AAIW_N_ enters the northern Tasman Sea but does not reach DSDP Site 593 and is distinguishable from AAIW_S_ in the δ^13^C of dissolved inorganic carbon (reflecting the addition of remineralized organic matter during AAIW_N_ transport within the subtropical gyre) [*Bostock et al*., 2004]. An increased presence of AAIW along the Chilean margin during glacial stages has been linked to a northward shift of the ACC with a potential contribution from increased AAIW production in the Southeast Pacific [*Martínez‐Méndez et al*., 2013], yet during the LGM, the southward extent of AAIW_N_ to the Tasman Sea was reduced [*Bostock et al*., 2004]. There is no overlap in glacial stage benthic δ^13^C_*P*.*wuellerstorfi*_ between DSDP Site 593 and the Chilean margin over the last 1 Ma (Figure 4), suggesting that DSDP Site 593 was not bathed by the AAIW that formed in the Southeast Pacific. In the late Pliocene, increasing sand content at DSDP Site 590B (1308 m water depth) from 3.5 Ma was interpreted to reflect an increasing northward influence of AAIW in the Tasman Sea [*Karas et al*., 2011a]. Although the record does not extend to the present day, the Pliocene increase in northward AAIW to DSDP Site 590 suggests that AAIW_S_ already had influence to the north of DSDP Site 593 by the late Pliocene. Furthermore, at present there is little difference between the temperatures of AAIW_N_ and AAIW_S_ [*Bostock et al*., 2004]. Thus, variable contributions from AAIW_N_ and AAIW_S_ in the Tasman Sea are unlikely to account for the observed IWT changes at DSDP Site 593, although further work is required to fingerprint the signatures and pathways of AAIW in the Pacific through the Pliocene‐Pleistocene.

Our benthic foraminifera data indicate that DSDP Site 593 has likely been bathed by AAIW throughout the Pliocene‐Pleistocene, as at present (Figure 1), and that our reconstructed IWT data therefore reflect AAIW temperature. We interpret our reconstructed AAIW properties as a reflection of conditions in the AAIW source regions, closely associated with the Subantarctic Front, including Antarctic Surface Water (AASW) properties, winter convection, and air‐sea buoyancy fluxes [*Hartin et al*., 2011; *Sloyan and Rintoul*, 2001]. These processes can lead to interbasin differences in AAIW properties: for example, to the south of Australia there is deep winter mixing and cooling of (warm, salty) Indian Ocean‐sourced SAMW as well as an addition of cold and fresh AASW [*McCartney*, 1977; *Sloyan and Rintoul*, 2001]. Using benthic foraminiferal δ^18^O and δ^13^C profiles from south of Tasmania, *Lynch‐Stieglitz et al*. [1994] identified a reduced contribution of Indian Ocean waters to AAIW during the LGM. Regardless of whether a Mg/Ca_sw_ correction is applied, the overall decrease in Mg/Ca_Uvigerina_ and monotonic increase in δ^18^O_P.wuellerstorfi_ over the last 3.5 Ma at DSDP Site 593 (Figure 2), across an interval of increasing continental ice volume [*Lisiecki and Raymo*, 2005; *Rohling et al*., 2014], is consistent with an overall shift toward cooler and fresher AAIW since the Pliocene. To fully understand how the Pliocene‐Pleistocene ocean density structure evolved will require development of water column profiles for the southwest Pacific incorporating benthic foraminiferal Mg/Ca and δ^18^O data with orbital‐scale resolution. Here we draw on the LGM as an analogue to interpret lower AAIW temperatures as a reflection of cooler and/or increased AASW contributions to AAIW [*Bostock et al*., 2004; *Lynch‐Stieglitz et al*., 1994], reflecting more vigorous winds, Antarctic sea ice expansion, and/or reduced contributions from warmer end‐members [*Lynch‐Stieglitz et al*., 1994; *Wainer et al*., 2012].

### 5.2 Implications for Pliocene‐Pleistocene Climate Evolution

#### 5.2.1 Pliocene‐Pleistocene Transition

On a global scale, the Pliocene‐Pleistocene transition centered on 2.7 Ma is marked by pronounced cooling in high‐latitude regions and upwelling regimes, decreasing atmospheric CO_2_, and increasing continental ice volume [*Lisiecki and Raymo*, 2005; *Martinez‐Boti et al*., 2015; *Martinez‐Garcia et al*., 2010; *Rohling et al*., 2014]. The new reconstructed mean and warmest Pliocene SSTs at DSDP Site 593 lie above the multimodel ensemble means for warm stages (14–16°C) at 40°S [*Dowsett et al*., 2012] and above modern SSTs. This occurs as both the weak Walker circulation [*Brierley and Fedorov*, 2010] and the relatively open Indonesian throughflow [*Karas et al*., 2011b] are expected to have reduced the strength of the East Australian Current [*Karas et al*., 2011a; *Lee et al*., 2002], suggesting that Pliocene warmth at DSDP Site 593 reflects proximity to the expanded warm pools rather than enhanced poleward heat transport.

We inferred (section 5.1) that the late Pliocene STF sat in a similar position to modern, potentially displaced equatorward by a few degrees latitude. In contrast, our Pliocene AAIW temperatures indicate warmer surface waters associated with the Subantarctic Front. Opal deposition in the Bellingshausen Sea [*Hillenbrand and Fütterer*, 2001] and diatom assemblages at multiple sites associated with the ACC [*Barron*, 1996a, 1996b] also demonstrate warmer surface ocean conditions, reduced sea ice cover, and a poleward displacement of the Antarctic Polar Front by 6° relative to present. In combination, these patterns suggest that a warmer and more latitudinally extensive subantarctic zone (between the STF and SAF) developed in the southwest Pacific sector of the Southern Ocean during the late Pliocene [*Ballegeer et al*., 2012]. This hypothesis requires further testing, however, since Ross Sea diatom assemblages indicate development of cooler surface ocean conditions with more persistent sea ice in the late Pliocene [*Riesselman and Dunbar*, 2013], which might lead to northward displacement of the SAF, and there is potential for bathymetric control over the position of the Antarctic Polar Front to the south of New Zealand [*Barron*, 1996b].

The late Pliocene cooling recorded at DSDP Site 593 in both SSTs (from 3.1 Ma) and IWTs (from 3.3 Ma) highlights the development of cooler subantarctic waters and/or northward displacement of the STF (section 5.1; Figure 6). Cooling and freshening of subantarctic surface waters from 3.5 Ma are also recorded by subsurface‐dwelling foraminifera, which record SAMW properties, at DSDP Site 590B (Figure 5) [*Karas et al*., 2011a]. At the same time, an increasing northward influence of AAIW at DSDP Site 590 also indicates evolving surface ocean conditions in the subantarctic region [*Karas et al*., 2011a]. From ~3.2 Ma surface ocean cooling also develops in the Subantarctic Atlantic (Figure 5) [*Martinez‐Garcia et al*., 2010] and in the Ross Sea [*Riesselman and Dunbar*, 2013]. A potential intensification and persistence of summer sea ice are recorded in the Ross Sea by ~3.03 Ma [*Riesselman and Dunbar*, 2013] and inferred from reduced biogenic opal deposition rates in the Bellingshausen Sea after ~3.1 Ma [*Hillenbrand and Fütterer*, 2001]. Development of a more extensive Antarctic ice sheet between 3.15 and 2.75 Ma [*Woodard et al*., 2014] also indicates changing climate conditions in the high latitudes of the Southern Hemisphere through the late Pliocene.

The culmination of the late Pliocene cooling at DSDP Site 593 at ~2.65 Ma in both SST and IWT is followed by a short interval of increased orbital‐scale variability in both records until ~2.4 Ma. The temperature minima at ~2.65 Ma are tentatively assigned to MIS G2, but this should be treated with caution given the low resolution of the benthic δ^18^O_*P*.*wuellerstorfi*_ data presented here (Table 1). The cooling begins earlier in IWT, from MIS G6 (~2.7 Ma). An abrupt decrease in deep South Atlantic benthic δ^13^C_*P*.*wuellerstorfi*_ at 2.75 Ma (Figure 4) has been attributed in part to more extensive sea ice and stratification around Antarctica [*Hodell and Venz‐Curtis*, 2006] and falls within a broader window of glacial stage cooling (2.7–2.4 Ma, MIS G6 through MIS 95) identified in other ocean basins in the late Pliocene [*Herbert et al*., 2010; *Lawrence et al*., 2011; *Naafs et al*., 2010; *Rohling et al*., 2014]. Reconstructed atmospheric CO_2_ concentrations highlight MIS G10 (~2.8 Ma) as the first time that a 275 µatm threshold for glaciation was crossed, with even lower concentrations recorded during MIS G6, G2 and 100 [*Martinez‐Boti et al*., 2015]. The temperature trends identified at DSDP Site 593 thus support other evidence for high‐latitude cooling in the late Pliocene, broadly associated with a decrease in atmospheric CO_2_.

Immediately after 2.65 Ma, both SST and IWT record warm interglacial maxima at DSDP Site 593, with values similar to those of the Pliocene (Figure 2). Particularly low inputs of glacial sediment to ODP 1119, east of New Zealand (Figure 1), at this time indicate a less extensive ice cap on the South Island than during the Pliocene [*Carter et al*., 2004] and support the evidence for regional warmth in the southwest Pacific. Relatively warm interglacials at ~2.5 Ma are also recorded in the Subantarctic Atlantic (Figure 5) [*Martinez‐Garcia et al*., 2010] and by two short‐lived increases in seasonal sea ice‐tolerant diatom taxa in the Ross Sea [*McKay et al*., 2012]. Thus, despite an overall transition toward globally cooler climate across the Pliocene‐Pleistocene boundary and INHG, surface ocean conditions in the Southern Ocean were highly variable and include intervals of relative warmth.

#### 5.2.2 Early Pleistocene Warmth

Between ~2.4 and 2.1 Ma, SSTs at DSDP Site 593 warm by ~3°C (400 kyr mean), then stabilize until ~.8 Ma (Figure 3). A similar but smaller (~1°C) warming is also observed at ODP Site 1125 toward 2 Ma [*Fedorov et al*., 2015] (Figure 5). Between 2.1 and 1.8 Ma, SSTs at DSDP Site 593 exceed the modern mean annual value and are comparable to all but the coldest stages of the Pliocene (Figure 2). This unusual early Pleistocene warmth highlights a strong regional control, consistent with a southward displacement of the STF and/or enhanced poleward heat transport into the Tasman Sea. Both scenarios contrast with the inferred equatorward migration and intensification of Hadley circulation cells, the Southern Hemisphere westerly wind belts, and polar water masses through the Pliocene‐Pleistocene [*Brierley and Fedorov*, 2010; *Martinez‐Garcia et al*., 2010; *Martinez‐Garcia et al*., 2011; *Rosell‐Melé et al*., 2014]. An alternative explanation for the early Pleistocene warmth at DSDP Site 593 is that the continued intensification of meridional temperature gradients through 3.5–2.0 Ma may have remained conducive to poleward heat transport [*Brierley and Fedorov*, 2010] via the East Australian Current. To test these hypotheses requires additional data from cores spanning the modern STF and subtropical regions of the southwest Pacific for the early Pleistocene.

#### 5.2.3 Middle and Late Pleistocene Evolution

A rapid SST decrease at 1.8 Ma marks the onset of long‐term surface ocean cooling at DSDP Site 593, coeval with evidence for evolving tropical and subtropical climate changes, including intensification of Walker circulation and subtropical upwelling [*Brierley and Fedorov*, 2010; *Ravelo et al*., 2004], and particularly strong glacial‐stage cooling in several tropical SST records (e.g., ODP Sites 662, 722, and 846) at 2.1 and 1.7 Ma [*Herbert et al*., 2010]. In contrast, most midlatitude and high‐latitude SST records show gradual cooling developing later (after ~1.6 Ma) and intensifying from 1.2 Ma in association with the MPT [*McClymont et al*., 2013], in line with the cooling we observe in IWT from 1.3 Ma. A tropical/subtropical control over the DSDP Site 593 surface cooling trend would imply a reduced heat transport by the East Australian Current, whereas the strengthening Walker Cell Circulation from 2 Ma [*Brierley and Fedorov*, 2010; *Fedorov et al*., 2015] ought to have the opposite effect. Cooling “upstream” in the tropical/subtropical source regions is also unable to explain the DSDP Site 593 SST trend, since SSTs in the West Pacific Warm Pool and Coral Sea remain stable or warm slightly (<1°C) between 2.0 and 1.0 Ma (Figure 5) (see discussion by *McClymont et al*. [2013]).

Surface ocean cooling from 1.8 Ma is also observed at ODP Sites 1125 and 1090 (Figure 5), the latter linked to a northward displacement of subpolar waters in the Subantarctic Atlantic [*Becquey and Gersonde*, 2002; *Martinez‐Garcia et al*., 2010]. We interpret the SST cooling in the southwest Pacific to reflect an increasing presence of subantarctic waters and northward displacement of the STF. The onset of IWT cooling, from ~1.3 Ma at DSDP Site 593, occurs within a broader window (from 1.5 Ma) of sustained low SSTs at DSDP Site 593 (Figure 2), intensification of cooling in ODP Site 1090 SSTs [*Martinez‐Garcia et al*., 2010], establishment of the modern high opal deposition belt in the Southern Ocean [*Cortese et al*., 2004], and a strong reduction in southern sourced water to the South Atlantic consistent with increased sea ice cover and/or surface ocean stratification in the Southern Ocean [*Hodell and Venz‐Curtis*, 2006]. Thus, the SST and IWT data from DSDP Site 593 confirm that climate evolution since 1.8 Ma was not restricted to the tropical or subtropical oceans but also affected the midlatitude and high latitude, first in association with the STF (DSDP Site 593 SSTs) and subantarctic waters (ODP Site 1090) [*Martinez‐Garcia et al*., 2010], and later in association with the SAF (DSDP Site 593 IWT).

*Martinez‐Garcia et al*. [2010] proposed that the coincidence of expanding subpolar waters in the Subantarctic Atlantic and cooling in the equatorial Pacific cold tongue from 1.8 Ma could be mechanistically linked via strengthening Hadley circulation, in response to intensification of the meridional temperature gradients. The new orbital resolution SST data from DSDP Site 593 confirms that the meridional temperature gradient in the southwest Pacific also intensified from 1.8 Ma; the cooling is larger than at ODP Site 1125 [*Fedorov et al*., 2015], but this may reflect differing sampling resolution (Figure 5) and/or the effect of bathymetric pinning of the STF at ODP Site 1125 discussed above (section 5.1). The relatively minor, and delayed, cooling, which occurs in IWTs as the surface ocean cools, suggests that before the MPT, the propagation of high‐latitude temperature signals to the low‐latitude regions via intermediate waters [*Lee and Poulsen*, 2008] is a less plausible teleconnection than via strengthening Hadley circulation since ~1.8 Ma [*Martinez‐Garcia et al*., 2010]. However, it is important to note that the magnitude of long‐term cooling in upwelling regions over the MPT (2–3°C) [*McClymont et al*., 2013] is comparable to that recorded in IWTs since ~1.5 Ma (almost 3°C; Figure 2). Understanding the relative influence of upwelling intensification, thermocline shoaling, and cooling of source waters may help to better constrain the factors driving the observed trends in upwelling sites and their utilization in calculations of meridional temperature gradients.

From 1.1 Ma, the amplitude of glacial‐interglacial SST variability at DSDP Site 593 increased [*Kender et al*., 2016], and a long‐term cooling trend develops between 0.9 and 0.6 Ma. Increased SST variability is also recorded at ODP Site 1090 in the south Atlantic but without any long‐term trend [*Martinez‐Garcia et al*., 2010]. The SST cooling at DSDP Site 593 after 0.9 Ma suggests a final intensification of the meridional temperature gradient during the MPT in the southwest Pacific. This contrasts with the largely stable meridional temperature gradient after ~1.2 Ma indicated at ODP Site 1090 [*Martinez‐Garcia et al*., 2010]. IWTs at DSDP Site 593 also indicate secular cooling from ~1.3 Ma, but this trend continues to the present day and is marked by an unusual pattern of reduced amplitude IWT oscillations after the MPT (~0.8 Ma) driven by a stepped decrease in interglacial maxima. It is unclear which processes explain this fall in interglacial IWTs, but during the late Pleistocene, increased production and/or deepening AAIW is recorded during millennial‐scale Antarctic warming events on the Chatham Rise [*Pahnke and Zahn*, 2005], with warmer AAIW at both Chatham Rise and DSDP Site 593 [*Elmore et al*., 2015]. *Pahnke and Zahn* [2005] attributed this relationship to reduced northward Ekman transport, in response to relaxation and southward displacement of the circumpolar wind systems. However, there is no shift in Subantarctic Atlantic dust flux at 0.8 Ma to suggest displaced/intensified westerlies [*Martinez‐Garcia et al*., 2011]. In the absence of detailed information from the Indian and Pacific sectors of the Southern Ocean spanning the MPT, the Atlantic data do not support a poleward displacement of the SAF to explain the interglacial warmth in DSDP Site 593 IWTs. Additional records of AAIW properties across the MPT are required from different oceanographic basins to determine whether the cause of the reduced interglacial maxima in temperatures is a regional phenomenon.

## 6 Conclusions

Through the Pliocene and Pleistocene epochs, expansion of polar waters and contraction of the tropical warm pools are considered to be important factors for lowering global mean temperatures, strengthening atmospheric circulation, and affecting heat transport between low and high latitudes [*Brierley and Fedorov*, 2010; *Martinez‐Garcia et al*., 2010]. Here we address the relative paucity of temperature data from surface and intermediate‐depth waters of the midlatitude and high latitude of the Southern Hemisphere through analysis of DSDP Site 593 in the Tasman Sea, southwest Pacific. Given current debates around the potential impact of evolving Mg/Ca_sw_ on temperature signals recorded in foraminifera Mg/Ca ratios, we present both uncorrected and corrected data for IWTs. The overall timings and trends of IWT evolution are robust regardless of the correction applied, but absolute AAIW temperature values can be raised by as much as 5°C for the Pliocene.

We show that the Pliocene‐Pleistocene has a general cooling trend in both SSTs and IWTs at DSDP Site 593, but the patterns are complex and include shifts in orbital‐scale variability, and times of relative warmth. The Pliocene is warmer than modern in both data sets, but we infer that the subtropical front of the ACC was positioned close to DSDP Site 593 and thus equatorward relative to present. Cooling begins from ~3.3 Ma (IWT) and ~3.1 Ma (SST), with links to tropical/subtropical warm pool extent and the equatorward expansion of subpolar water masses in the Southern Ocean. Both SSTs and IWTs record marked cooling trends, which culminate at 2.65 Ma, and the start of a longer‐term cooling trends from 1.8 and 0.9 Ma (SST) and 1.3 Ma (IWT), coeval with cooling and ice sheet expansion noted in other regions associated with the Pliocene‐Pleistocene transition and the MPT. The early Pleistocene is marked by relatively warm SSTs, indicating increased contributions of subtropical surface waters to the southern Tasman Sea. The observed trends in SST and IWT are not identical despite both having an underlying link to the position and/or intensity of circulation within ACC. The results presented here demonstrate the importance of reconstructing and understanding the evolution of different sectors of the Southern Ocean, and the thermal history of both the sea surface and the ocean interior, in order to fully understand Pliocene‐Pleistocene climate evolution in the Southern Hemisphere.
